# Integrating Clinical Indexes into Four-Diagnostic Information Contributes to the Traditional Chinese Medicine (TCM) Syndrome Diagnosis of Chronic Hepatitis B

**DOI:** 10.1038/srep09395

**Published:** 2015-03-23

**Authors:** Hong Kang, Yu Zhao, Chao Li, Yujia Chen, Kailin Tang, Linlin Yang, Chao Ma, Jinghua Peng, Ruixin Zhu, Qi Liu, Yiyang Hu, Zhiwei Cao

**Affiliations:** 1 School of Life Sciences and Technology, Tongji University, Shanghai 200092, P.R.China; 2 Institute of Liver Diseases, Shuguang Hospital Affiliated to Shanghai University of Traditional Chinese Medicine, Shanghai 201203, P.R.China; 3 Key Lab of Systems Biology, Shanghai Institutes for Biological Sciences, Chinese Academy of Sciences, Shanghai 200031, P.R.China; 4 E-Institute of TCM Internal Medicine, Shanghai Municipal Education Commission, Shanghai 200003, P.R.China; 5 School of Biomedical Informatics, University of Texas Health Science Center, Houston. TX 77030, USA

## Abstract

Traditional Chinese Medicine (TCM) treatment has been commonly used to treat Chronic Hepatitis B (CHB) in Asian countries based on TCM syndrome diagnosis, also called “ZHENG”. The syndrome is identified through the four-diagnostic methods, with certain degree of subjectivity and ambiguity from individual doctors. Normally those CHB patients also receive series of parameters from modern clinical examination, while they are routinely believed to be unrelated with the TCM syndrome diagnosis. In this study, we investigated whether these biomedical indexes in modern medicine could be beneficial to TCM syndrome diagnostics in an integrative way. Based on 634 patient samples from health controls and three subtypes of CHB syndromes, a two-view based hierarchical classification model was tested for TCM syndromes prediction based on totally 222 parameters integrated from both TCM practice and modern clinical tests. The results indicated that the performance of syndrome classification based on a proper integration of TCM and modern clinical indexes was significantly higher than those based on one view of parameters only. Furthermore, those indexes correlated with CHB syndrome diagnosis were successfully identified for CM indexes and biochemical indexes respectively, where potential associations between them were hinted to the MAPK signaling pathway.

As an important complementary and alternative medication system, Traditional Chinese Medicine (TCM) based on syndrome diagnosis has long been used to treat Chronic Hepatitis B (CHB) disease in Asian countries especially in China^1^,^2^. TCM syndrome, also called “ZHENG” in Chinese, can be viewed as a summary of comprehensive signals from a particular stage of disease development such as the tongue color and pulse^3^. It is well known that accurate discrimination of the syndrome state is critical to the treatment of many chronic and systematic diseases, like the CHB disease, which presents to be a major global health problem with estimates of nearly 400 million people infected worldwide^4^,^5^. However, current TCM syndrome diagnosis is still highly experience-based rely on four-diagnostic methods. The four-diagnostic methods have been historically summarized based on logical reasoning and empirical experience, therefore syndrome differentiation from individual doctors is often determined with certain degree of subjectivity and ambiguity. Despite of the judging standard, there is often deviation on a specific patient when different doctors determine the syndrome, especially when the syndrome development is at the early stage or transition state (JIAN-ZA ZHENG). As a major scientific challenge of CM study, objective and quantitative syndrome diagnosis are highly desirable to improve the efficacy of CM treatment.

In reality, the same patient seeking TCM treatment also receives a list of biochemical indicators from modern clinical examination. Such biomedical indexes from modern medicine, often taken as more objective and quantitative than four-diagnosis parameters, are generated to describe the same disease state for the same HBV patient while from different perspective. Both set of parameters are important references to disease diagnosis and efficacy assessment. Considerable achievements have been made in the correlation of CM syndromes differentiation with measurable modern biomedical indexes in modern medicine and more are in progress^6^. Recently Wang et al.^7^ tried to combine clinical indexes with the four-diagnostic information for TCM syndrome diagnosis in liver cirrhosis samples. Six classification models were exploited to select the top parameters from both sets of indexes. As the only reported model combining both sets of indicators to date, the investigation is highly informative, but whether the clinical indicators contribute to diagnosis of syndrome has not been fully explored yet.

Intuitively, both clinical and “four-diagnostic” indexes are indicators of the same patients at the same disease stage. They can be considered as two sets of independent profiles of different views for the same patients. With properly integration, they might benefit each other to improve the TCM syndrome diagnosis. Based on such assumption, we tested a multi-view learning based strategy for CHB syndrome diagnosis by integrating both sets of indexes hierarchically. The basic idea of this strategy is that, when each view of features provides independent perspective for the sample description, proper integration of them will eventually lower the likelihood of biased judgment when comparing to the strategy of single view^8^. We believed that exploring the complementarity as well as the correlation and potential mechanism between these two indexes have significant meaning in syndrome differentiation.

Our study was performed on a large cohort of totally 634 patient samples collected in china, including 29 healthy controls and 605 CHB cases with three commonly observed syndromes: Liver-Gallbladder Dampness-Heat (LGDH, 363 patients), Liver-Stagnation Spleen-Deficiency (LSSD, 193 patients) and No Distinct Symptom (NDS, 49 patients). In TCM systems, LGDH and LSSD are the most abundant syndromes of CHB patients^9^. For instance, patients with typical LGDH symptoms are normally featured as yellow tongue, pale yellow complexion, fetid mouth odor and bitter taste in mouth, while those with LSSD are often described as pale white tongue, depression, impatience and abdominal distension etc.^10^. NDS is also frequently observed in TCM clinics to describe those patients with no distinct TCM symptoms while infected with HBV viruses already. In our study, each of these samples is provided with two independent sets of indexes: 108 features from four-diagnostic information and 114 features from modern clinical tests. Each of the patients was labeled by one syndrome class. A specialized statistical learning model was presented to investigate the contribution of the modern clinical tests data to the TCM syndrome classification based on a multi-view strategy. The putative mechanism linking the two views of indexes was hinted as well.

## Results and Discussion

### Independency exists between TCM and modern clinical index

**Figure 1:**
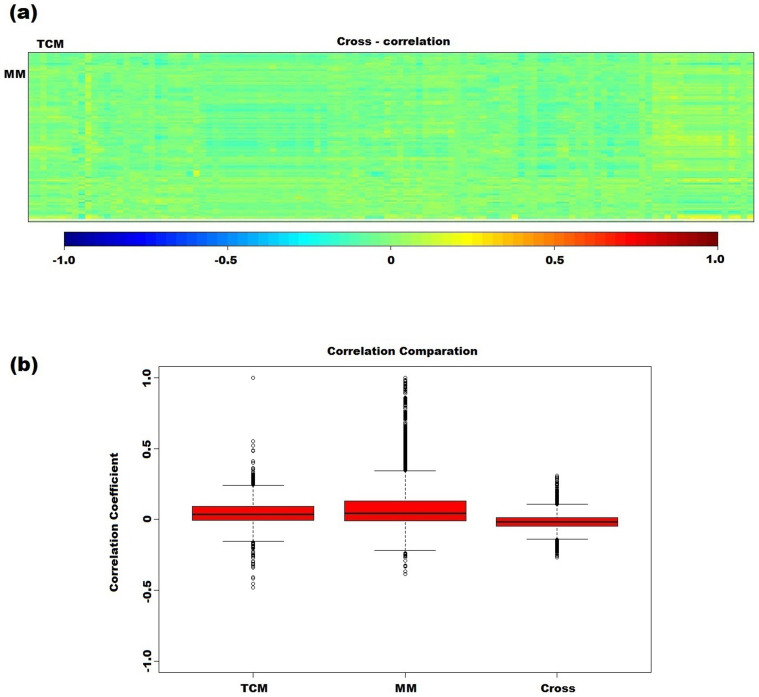
Correlation analysis between the features of TCM and MM. (a) Correlation matrices between TCM indexes and MM indexes. Correlation values are represented in heat map (blue: negative correlation; red: positive correlation). (b) Box plot distribution of feature correlation within (the first two box plots) and between (the last box plot) TCM and modern medicine (MM) indexes.

To investigate the dependency between these two profiles of the same datasets, Canonical Correlation Analysis (CCA, see *Methods*) was performed to link the 108 TCM features and 114 clinical features of the 634 samples (Figure 1(a)). As shown in Figure 1, the rectangular 114 × 108 matrix presents the correlations between the variables of TCM and modern medicine (MM), and no significant correlations were observed among them. By contrast, the correlation coefficients within each group of TCM indexes or modern clinical tests are much higher than inter-group (Figure 1(b)). For instance, two clinical indexes TBIL and DBIL were highly correlated (correlation coefficient 0.938). The high independency and complementarity between the CM and MM indexes inspire us to adopt a multi-view strategy to classify TCM syndromes.

### A hierarchical co-training model for TCM syndrome diagnosis

#### Model selection

In previous study, six in-silico models were tested and only two models gained improvement by integrating modern clinical indexes^7^. Their results indicated that the contribution of modern clinical data is closely related to model selection, while they only used the samples with clearly labeled information for classifications. We noticed that in TCM syndrome diagnosis, the large sets of unlabeled data, such as those patient samples hard to be assigned, or yet developing to a typical syndrome category are substantially distributed and waiting to be explored. Such unlabeled data contain potential value for classification. Taking advantages of unlabeled data for supervised classification is often categorized to the semi-supervised learning methods^11^. In this case, a specialized semi-supervised based co-training model was adopted based on a multi-view strategy, where unlabeled data was taken fully consideration for TCM syndrome diagnosis.

#### Model construction

Analysis of the four labels of TCM syndrome indicated that they can be classified in a hierarchical way: The samples can be firstly classified into the CHB group or the healthy group, then the CHB samples can be further grouped into Distinct Symptom (DS) and No Distinct Symptom (NDS). Finally the DS group can be classified into LGDH and LSSD. Therefore in our study, three sub-models were established separately using the co-training based random forest method to classify these cases respectively. (See *Methods*).

**Figure 2:**
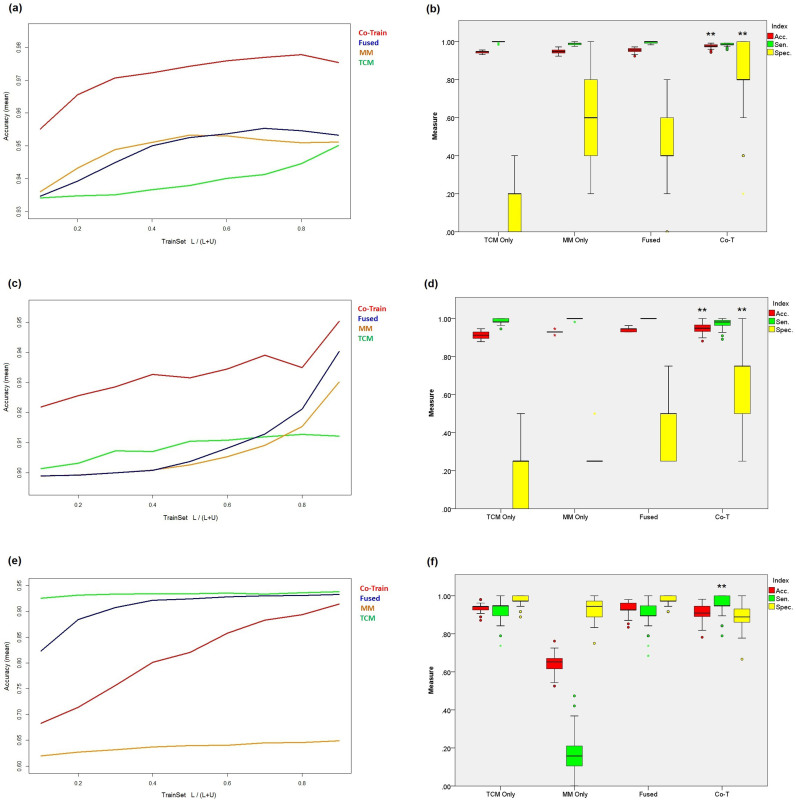
Evaluation of the co-training algorithm. (a), (c) and (e) present the average accuracy of the differentiation models on CHB vs. health controls, DS vs. NDS and LGDH vs. LSSD respectively, with the percentage of training data increased from 10% to 90% at an interval of 10%. (b), (d) and (f) present the distribution of accuracy, sensitivity and specificity for individual and combined view of TCM and MM, as well as the co-training model on each syndrome differentiation. (**p < 0.001, *p < 0.01, random times = 100).

As a result, Figure 2(a) presents the average accuracy when the percentage of training data was adjusted from 10% to 90% at 10% interval in the first sub-model, and compared with the single TCM view model, single MM view model and the simple combined view model respectively (see *Methods*). The accuracies for the four models all raised with the increasing of the training data, among which the accuracy of co-training model is remarkable higher than those of others. The accuracy of the single TCM view model is slightly lower than those of single MM view model and the combined one. This is not surprising, since the clinical index might diagnose CHB from health controls more precisely than that of TCM.

Similarly, the second sub-model was trained to classify the CHB group into DS and NDS. As the increasing of the percentage of training data, the accuracy of the four models all raised (Figure 2(c)). The performance of co-training was also the best among others.

Figure 2(e) and Figure 2(f) present the classification results for the four models in distinguishing LGDH and LSSD. It should be noted that although the co-training model achieved the best sensitivity among others, its accuracy is lower than the single TCM view model, which was quite different from the scenarios of the first two sub-models. This is probably due to that the TCM indexes are dominant compared to MM indexes in the distinguishing of two TCM syndromes LGDH and LSSD from the DS.

In summary, Table 1(a) presents the overall evaluation results of these models. Comparing with that of single view of TCM, the MM view model performed well not only in the differentiation of CHB vs. health control, but also in that of the DS vs. NDS. Although we would expect that single view TCM model differentiate DS vs. NDS well, such results indicates that the biochemical information from modern medicine actually contains potential value for the TCM syndrome diagnosis and may be further integrated with TCM information.

#### An integrative predictor for CHB syndrome diagnosis

**Figure 3:**
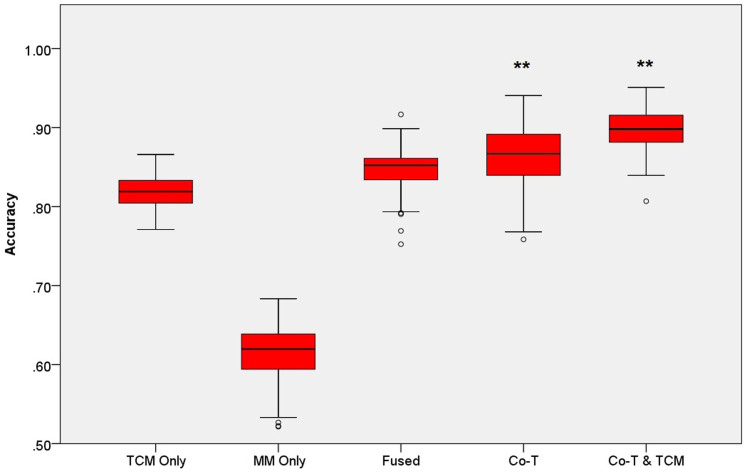
The comparison of the prediction performances of five models on the differentiation of CHB syndromes. (**p < 0.001, *p < 0.01, random times = 100).

Based on our trained sub-models, we present a pure black-box predictor for TCM syndrome diagnosis without any prior information, which should be helpful in CHB syndrome diagnosis. The first two sub-model of this predictor rely on co-training model since the average accuracy of the co-training model is highest (0.8692). Considering the outstanding accuracy of the single TCM view model on differentiation of LGDH vs. LSSD, we use it in the third sub-model instead of co-training, and found that the accuracy of the whole model reached to 0.8995. Therefore, we used a co-train model for the first two level classification and a single TCM view model for the last level classification among the whole hierarchical classification, and termed the final black-box predict as “co-training & TCM” for short, as shown in the related figures and tables (Figure 3 and Table 1(b)).

### Potential mechanisms linking two views of CHB syndrome diagnosis

#### Feature selection for TCM syndrome diagnosis

**Figure 4:**
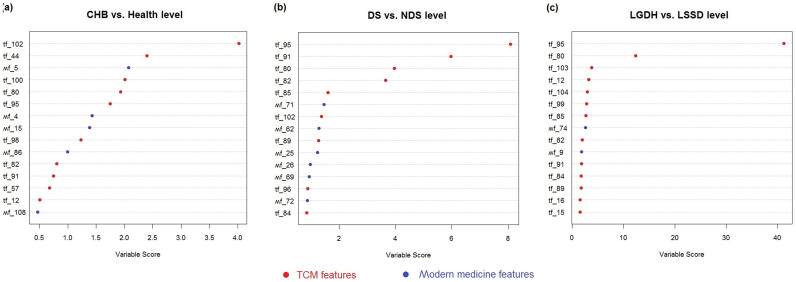
The factor importance for each sub-model. The top 15 factors which were selected by the co-training model are presented, and highlighted in red and blue to distinguish TCM and MM features.

In order to identify the key factors and evaluate the contribution of the TCM and MM features on the syndromes diagnosis for all the three differentiation cases, i.e., CHB vs. healthy group, DS vs. NDS, and LGDH vs. LSSD respectively, feature selection was performed (see *Methods*). For each sub-model, Figure 4 presents the top 15 important features which are highlighted by red and blue, indicating that they are from TCM or MM respectively. For the differentiation of CHB vs. health control, there's no significant difference between the contributions of the two views. Within the top 10 features, 6 is from TCM and 4 is from clinical indexes (Figure 4(a)). For the other two classifiers, TCM is the dominant view with 7 and 8 important features among the top 10 variables respectively (Figure 4(b) and Figure 4(c)). Nevertheless, we also identified several modern clinical indexes that help to improve the syndrome differentiation in TCM effectively.

From Figure 4 and Table 2, it can be seen that: The key TCM factors for CHB syndrome prediction mainly come from the characters of pulse manifestation, tongue and tongue coating. For example, comparing with healthy controls, the CHB patients have much more chance to get wiry pulse, feel fatigued, with red tongue and yellow tongue coating. The patients from DS group are more likely to get greasy fur, and the color of tongue and tongue coating is deeper than those of NDS group. While differentiating LGDH group and LSSD group, heavy yellow coating/red tongue and the bitter taste in mouth are found to be the most significantly difference.

On the other hand, several MM indices seem to play important roles in TCM syndrome differentiation for CHB diseases. For instance, the DS group has much higher APOA-1 and T_4_ than those of the NDS group. As a functional protein which promotes cholesterol efflux from tissues to the liver for excretion, APOA-1 was found to decrease obviously in the severe type B hepatitis and the chronic severe type B hepatitis^12^. Meanwhile, liver plays an important role in the synthesis and degradation process of thyroxine (T_4_)^13^. Furthermore, the LGDH group tends to have much higher basophilic granulocyte (%) and ALB than those of the LSSD group, which might be related to the level of liver dysfunction.

#### EGF and thyroxin might be involved in DS/NDS differentiation through influencing the color of tongue/tongue fur

**Figure 5:**
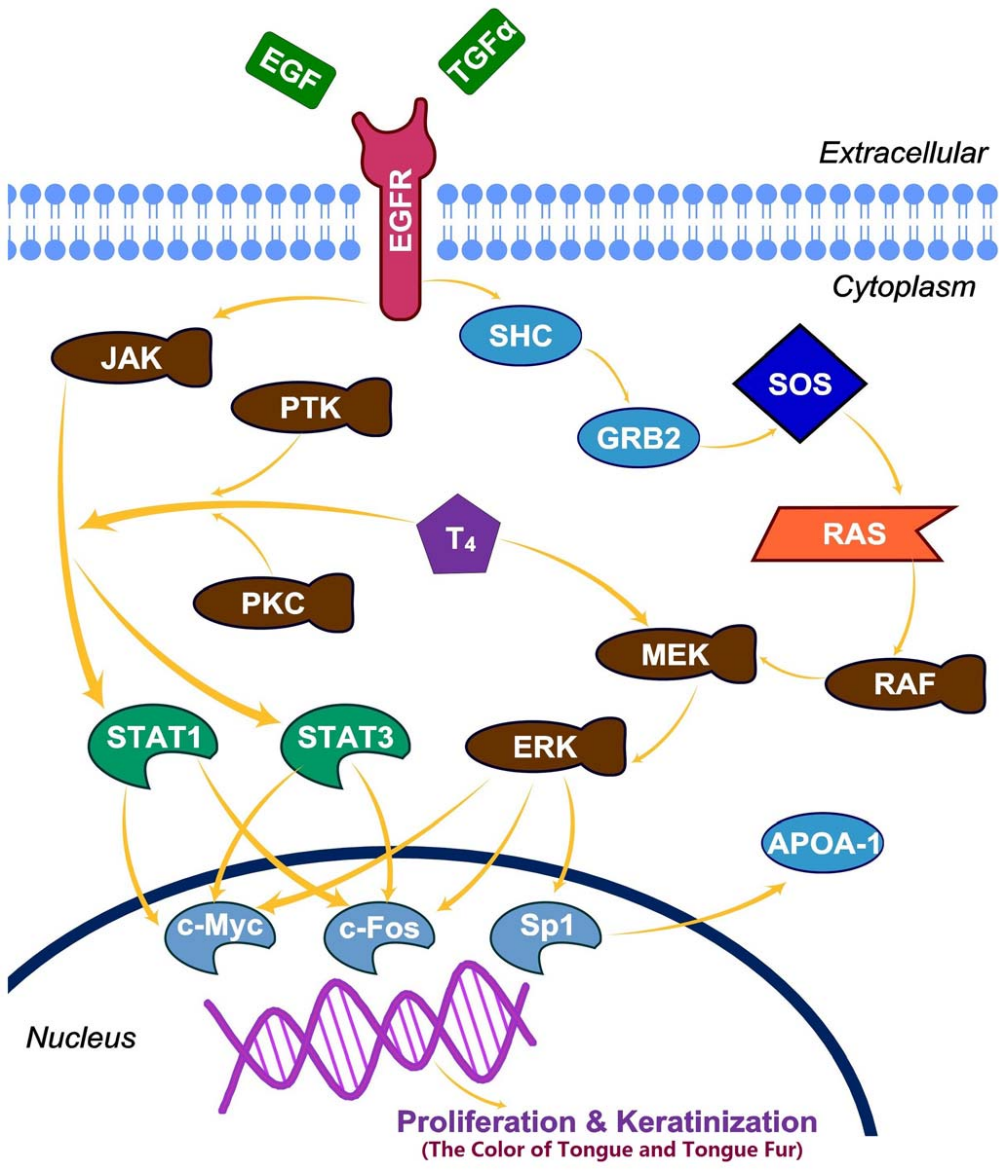
T_4_ potentiates the EGF induced cellular proliferation and keratinization. The proliferative signal of EGF is transmitted through two pathways: (1) JAK/STAT pathway and (2) Ras/MAPK pathway, ultimately causing activation of transcription factors such as c-Fos, STAT1 and STAT3, which all have important roles in proliferation and keratinization. T_4_ was reported to potentiate the EGF signaling through both the STAT pathway and the MAPK pathway.

To further explore the potential correlation between the TCM indices and MM indices, an extended signal pathway (Figure 5) was constructed based on literature investigation. Comparing to the Non-distinct-syndrome (NDS) patients, distinct syndrome (DS) group tends to have stronger tongue color, darker and greasier tongue fur, higher thyroxin and APOA-1 level. A comprehensive literature review found that, the color of tongue and tongue fur may closely related to epidermal growth factor (EGF) and transforming growth factor alpha (TGF-α). EGF/TGF-α could stimulate the intrinsic protein-tyrosine kinase activity, and initiates certain signal transduction cascade^14^ that probably regulate the proliferation and keratinization of epidermal cell on the dorsum of tongue. Then APOA-1 expression inducted by EGF was reported to be medicated by the Ras-MAP kinase cascade and Sp1^15^. Meanwhile, thyroxin/T4 was found to be able to potentiate the EGF signaling through the STAT pathway and the MAPK pathway respectively^16^. To summarize the above points, the underlying mechanism was proposed at a network level to explain the correlation between CM indexes and MM indexes, as shown in Figure 5. Based on this suggested mechanism, T4 would be positively correlated with APOA-1 level. So does to the T4 and the color of tongue/tongue fur.

A statistical analysis was carried out to test above proposal on the sample data of 556 DS patients. In clinical samples, T4 and APOA-1 were observed to increase simultaneously with a highly significant p-value of 2.78 × 10^−9^, Similarly, T4 and color of tongue/tongue fur were find to elevate simultaneously with p-value of 0.012. This observation supports our proposal well because when T4 increases, both APOA-1 and tongue/tongue fur color will be boosted.

Currently we are not able to suggest any concrete mechanism between bitter taste and basophilic granulocyte difference in LGDH and LSSD syndrome yet. What we may speculate might involve vitamin adsorption and immune response difference. The simple reason is that the bitter taste was reported to relate with the absorption of vitamin C and B_12_^17^, while APOA-1 is one of the most important enzymes in the pathway of vitamin absorption.

As a summary, in this paper, we adopted a hierarchical model co-trained by integrated features of TCM practice and modern clinical examinations to classify the HBV syndromes. Our results indicated that with appropriate model, CHB syndrome diagnosis can be improved remarkably than through other models focusing on only one class of features. In this sense, the modern clinical indexes can be useful to improve syndrome classification. Finally, the association between the two classes of significant indexes suggested that, MAPK pathway might be involved into the potential mechanism of syndrome development where EGF and thyroxin may influence the color of tongue and tongue coating. Our study help to bridge the gap between CM and MM indexes in differentiating CHB syndromes, so as to explore the mutual complementarity of the two set of indexes. Although our current study focuses on the contribution of clinical indices to TCM syndrome diagnosis, we believe that the patients' stratification strategy of TCM will be useful to design more effective clinical strategy in modern medicine area, with increasing scientific basis of TCM syndrome is revealed.

## Methods

### Data sources

634 patient samples were collected in the Shuguang Hospital (the affiliated hospital of Shanghai University of TCM), the Infectious Disease Hospital of Ningbo and the Sixth of People's Hospital of Shaoxing Zhejiang from October 2009 to October 2010 (Table 3). The whole samples were well curated and the CHB patients were selected based on the following criteria: 1) all patients were diagnosed according to both CHB and TCM syndromes, and confirmed by chief physicians in Chinese; 2) the diagnosis of CHB was based on the increased ALT levels (above the upper limit of the normal range) in at least two blood samples assayed over a 6-month period, and with the presence of detectable hepatitis B surface (HBs) antigen and (or) HBV DNA; 3) the TCM syndrome differentiation was referred to the viral hepatitis diagnostic standard that described by Internal Medicine Hepatopathy Committee of Chinese Traditional Medicine Association in December, 1991^18^.

In our dataset, each sample is represented with 108 features (Supplementary Table S1) from the four-diagnostic information of TCM. They are regarded as the basic symptoms required by physicians to identify the CHB syndromes in clinic. Also 114 clinical features (Supplementary Table S2) from clinical indexes were identified. Symptoms of TCM are encoded using the four-value ordinal scales to indicate their severity degree: 1 for no symptom, 2 for the normal level of the symptom, 3 for the medium serious level of the symptom, and 4 for the most serious level. Sign of TCM are encoded using the binary coding to indicate whether the patient has such sign or not, 0 for no sign and 1 otherwise. The proportion of missing value in TCM features and clinical indexes are 0% and 9.75% respectively. In order to reduce the influence of the missing values, they were imputed by mean values of the instances in the same field in the data preprocessing step.

Noted that there exist several different diagnostic criterion of Chronic Hepatitis B TCM syndrome. While no solid and well-accepted one existed. The ZHENG diagnosis criteria in this study was adopted according to the “Viral hepatitis TCM syndrome differentiation standard”^19^ and the “Guideline for Clinical study of new drugs in Chinese herbs”^20^. Those 108 TCM features, covering all the information involved by the two sets of syndrome diagnosis criterion, are recognized as closely related symptoms with liver diseases in TCM theory. MM indexes cover all common used factors in diagnosis and assessment of CHB^21^,^22^, such as the serum indicators of Hepatitis B virus markers (HBsAg, HBsAb, HBeAg, HBeAb, HBcAb, HBVDNA), liver and kidney functions, Immune parameters (CD3, CD4, CD8, IgA, IgM, IgG), hormone (T3, RT3, T4, T, E2), fibrosis (HA, LN, IV-C, P-III-P), cytokines (IL-1、 IL-2、 IFN、 TNF-a), tumor marker (AFP), lipids (TC, TG, HDL-C, LDL-C, APOA-1), blood glucose, serum globulins (albumin, α1-globulin, α2-globulin, β-globulin, γ-globulin), blood routine test etc.

### Canonical Correlation Analysis of two view indexes

Canonical Correlation Analysis (CCA)^23^, a multidimensional exploratory statistical method is commonly used to explore the sample correlations between two sets of quantitative variables observed on the same experimental units. For every pair of the features from the two views, CCA calculates a correlation coefficient which presents the details of the relationship between different views.

In this study, CCA was firstly employed to analyze the correlation between TCM indicators and clinical indexes of CHB patients. In the following study, *n* denotes the number of instances in our dataset (*n* = 634), *p* and *q* are the numbers of the features from the two views respectively (*p* = 108, *q* = 114), Classical CCA was adopt here rather than Regularized CCA, since *p* < *n* and *q* < *n*.

### Random forest based co-training classifiers

A co-training based strategy was used in our study to integrate the TCM and Modern Medicine (MM) information for TCM syndrome analysis. This model was first presented in machine learning community by Tom M. Mitchell et al.^24^. Recent applications indicate that co-training algorithm obtains strong performance in classification especially for multi-view data integrations^11^.

Basically, co-training is designed to handle the classification problem in which the sample features are obtained from diverse views and can be partitioned into disjoint set, while the class label is available for only a subset *m* out of the *n* total observations. In other word, we only have *m* labeled training samples while the remains are unlabeled with no class information^24^. The goal of co-training is to incorporate all the information available from the views together with all the *n* observations to accurately predict the class label of the remain *n*-*m* samples. Different from the naïve data fusion approaches which simply merge the features from different view together as a whole vector to fit the classifier, the co-training typically involves firstly training a classifier directly on each view, and then provides a mechanism to combine predictions across views into an overall estimate. In this study, the random forests (RF) procedure was adopted as the base classifier in the co-training schema due to its high efficiency. Detailed algorithm can be accessed in Supplementary Materials (Supplementary Text S1).

We formed the whole diagnosis into a hierarchical classification model (Supplementary Figure S1) and several techniques were adopted in this study:

1. To determine the best ratio of labeled training set, the percentage was set from 10% to 90% at an interval of 10%. For each percentage, the co-training algorithm was executed and evaluated with different training set for 100 times.
2. The co-training schema in the syndrome differentiation of CHB is compared with another three models, *i.e.*, the classification based on features from single view (TCM view or MM view respectively), and a simple aggregation way (combining the views of TCM and MM directly to create a whole vector representation). The accuracy, sensitivity and specificity were also calculated and averaged over 100 different training data for each percentage respectively.

All methods were carried out in accordance with the approved guidelines. This project was approved by Chinese Clinical Trial Registry (Registration Number: ChiCTR-DCC-10000759). Together with the second round clinical data collection in 2012, the study was approved by ethic committee: IRB of Shuguang Hospital affiliated with Shanghai University of TCM (Permit Number: 2012-206-22-01). An informed consent was signed by each of the participants, and the study protocol conformed to the ethical guidelines of the Declaration of Helsinki (1964).

## Author Contributions

Project planning and design: H.K., Q.L., Y.H. and Z.C. Data collection: Y.Z., J.P. and Y.H. Modeling: H.K., K.T., C.L. and L.Y. Pathway analysis: H.K., Y.Z., Y.C., C.M. and R.Z. Manuscript: H.K., Q.L. and Z.C.

## Supplementary Material

Supplementary Information Supplementary Data

## Figures and Tables

**Table 1:** (a) The performance comparisons of the co-training model with single and simple combined views of TCM and MM on each syndrome differentiation. (b) The prediction performance of five models on the differentiation of CHB syndromes

(a)				
	TCM only	MM only	Simple combination	Co-training
	Classifier 1 (Healthy vs. CHB)			
Acc.(SD)	0.9445 (0.006)	0.9509 (0.011)	0.9546 (0.010)	**0.9778 (0.011)
Sen.(SD)	0.9992 (0.003)	0.9891 (0.005)	0.9967 (0.005)	0.9842 (0.008)
Spec.(SD)	0.1400 (0.138)	0.5380 (0.232)	0.4500 (0.178)	**0.824 (0.197)
	Classifier 2 (NDS vs. DS)			
Acc.(SD)	0.9122 (0.016)	0.9301 (0.008)	0.9403 (0.011)	**0.9503 (0.025)
Sen.(SD)	0.9884 (0.013)	0.9984 (0.005)	1.0000 (0)	0.9714 (0.025)
Spec.(SD)	0.1600 (0.161)	0.2875 (0.090)	0.4150 (0.167)	**0.66 (0.215)
	Classifier 3 (LGDH vs. LSSD)			
Acc.(SD)	0.9385 (0.023)	0.6489 (0.044)	0.9329 (0.028)	0.9141 (0.040)
Sen.(SD)	0.9257 (0.046)	0.1742 (0.092)	0.9121 (0.067)	**0.9573 (0.049)
Spec.(SD)	0.9758 (0.027)	0.9299 (0.045)	0.9744 (0.023)	0.8913 (0.057)

(b)					
	TCM only	MM only	Simple combination	Co-T	Co-T & TCM
Acc.(SD)	0.8167 (0.021)	0.6095 (0.034)	0.8467 (0.025)	**0.8692 (0.039)	**0.8995 (0.031)

**p < 0.001, *p < 0.01; Random times = 100.

TCM: Traditional Chinese Medicine; MM: Modern Medicine.

Acc: Accuracy; Sen: Sensitivity; Spec: Specificity.

CHB: Chronic Hepatitis B.

LGDH: Liver-Gallbladder Dampness-Heat.

LSSD: Liver-Stagnation Spleen-Deficiency.

NDS: No Distinct Symptom.

**Table 2:** The factor importance for each sub-classifier. For each of them, 10 features which contribute most to the model are presented and evaluated. “tf” and “mf” stand for the TCM and MM feature respectively, and the numbers in brackets are standard deviations. 1) the value of variable “tongue color” from 1 to 6 represent pale, light red, red, crimson, light purple and dark purple respectively; 2) the value of “tongue fur color” from 1 to 6 represent white, yellowish, yellow, deep yellow, grey and dark. 3) For the binary attribute of TCM, if the value is 1, it means the patient has such symptom, and 0 otherwise. 4) For the categorical attributes of TCM, taking feeble pulse/replete pulse as an example, −1 for feeble pulse, 1 for replete pulse and 0 for normal control

Classifier 1 (Healthy vs. CHB)					
Rank	Feature	Name	Healthy	CHB	P-value
1	tf_102	Wiry pulse	0.10 (0.31)	0.66 (0.47)	7.123e−11
2	tf_44	Lack of strength	1.03 (0.19)	1.49 (0.56)	6.325e−16
3	mf_5	AST (IU/L)	19.77 (5.06)	43.48 (31.26)	<2.2e−16
4	tf_100	Feeble pulse/replete pulse	0.17 (0.38)	0.01 (0.13)	0.031
5	tf_80	Tongue color	2.31 (0.97)	2.78 (1.17)	0.03576
6	tf_95	Tongue coating color	1.24 (0.51)	1.88 (0.84)	2.279e−07
7	mf_4	ALT (IU/L)	19.8 (7.68)	79.77 (89.4)	<2.2e−16
8	mf_15	HBsAg (IU/L)	0.01 (<0.01)	233.29 (55.8)	<2.2e−16
9	tf_98	Floating pulse/Deep pulse	0.24 (0.44)	0.06 (0.28)	0.03535
10	mf_86	Mean platelet volume (fL)	8.17 (0.96)	10.43 (1.56)	5.895e−14

Classifier 2 (NDS vs. DS)					
Rank	Feature	Name	NDS	DS	P-value
1	tf_95	Tongue coating color	1.04 (0.20)	1.96 (0.84)	<2.2e−16
2	tf_91	Greasy fur/Curdy fur	0 (0)	−0.53 (0.51)	<2.2e−16
3	tf_80	Tongue color	2.02 (0.32)	2.84 (1.19)	<2.2e−16
4	tf_82	Lustrous tongue/Withered tongue	−0.98 (0.14)	−0.63 (0.54)	<2.2e−16
5	tf_85	Teeth-marked tongue	0.04 (0.20)	0.24 (0.43)	6.756e−08
6	mf_71	IV-C (ug/L)	55.8 (6.1)	55 (11.98)	0.8518
7	tf_102	Wiry pulse	0.55 (0.50)	0.67 (0.47)	0.07962
8	mf_62	APOA-1 (g/L)	0.98 (0.19)	1.24 (0.31)	9.089e−12
9	tf_89	Thin fur/Thick fur	0 (0)	0.27 (0.49)	<2.2e−16
10	mf_25	Thyroxine, T4 (nm/L)	111.2 (24.3)	124.7 (28.4)	0.1562

Classifier 3 (LGDH vs. LSSD)					
Rank	Feature	Name	LGDH	LSSD	P-value
1	tf_95	Tongue coating color	2.44 (0.61)	1.05 (0.21)	<2.2e−16
2	tf_80	Tongue color	3.31 (1.05)	1.95 (0.91)	<2.2e−16
3	tf_103	Thready pulse	0.10 (0.30)	0.40 (0.49)	1.476e−13
4	tf_12	Bitter taste in mouth	1.51 (0.65)	1.11 (0.32)	<2.2e−16
5	tf_104	Slippery pulse	0.25 (0.43)	0.04 (0.19)	3.762e−15
6	tf_99	Slow pulse/Rapid pulse	0.21 (0.41)	0.03 (0.23)	1.301e−10
7	tf_85	Teeth-marked tongue	0.11 (0.31)	0.49 (0.50)	<2.2e−16
8	mf_74	Basophilic granulocyte (%)	0.41 (0.27)	0.33 (0.26)	0.001954
9	tf_82	Lustrous tongue/Withered tongue	−0.69 (0.54)	−0.53 (0.51)	0.0011
10	mf_9	ALB (g/L)	34.29 (7.38)	31.69 (7.03)	7.333e−05

**Table 3:** The sample percentages of each TCM syndrome

**Syndrome**	LGDH	LSSD	NDS	Healthy
**Sample size**	363	193	49	29
**Percentage**	57.3%	30.4%	7.7%′	4.6%
