# Supplementary material for: Integrating Clinical Indexes into Four-Diagnostic Information Contributes to the Traditional Chinese Medicine (TCM) Syndrome Diagnosis of Chronic Hepatitis B
Source: Sci Rep. 2015 Mar 23;5:9395. doi: 10.1038/srep09395 (PMC4369723; doi:10.1038/srep09395)
Supplement: Supplementary Information — Supplementary Data [file srep09395-s1.doc]

**Integrating Clinical Indexes into Four-Diagnostic Information Contribute to the Traditional Chinese Medicine (TCM) Syndrome Diagnosis of Chronic Hepatitis B**

Hong Kang, Yu Zhao, Chao Li, Yujia Chen, Kailin Tang, Linlin Yang, Chao Ma, Jinghua Peng, Ruixin Zhu, Qi Liu, Yiyang Hu and Zhiwei Cao

***Supplementary Data***

**Table S1A**. Four-Diagnostic Information in TCM.

| **Index** | **Feature** | **Data range** |
| --- | --- | --- |
| 1 | Headache(头痛) | Four-level scale |
| 2 | Dizzy(头晕) | Four-level scale |
| 3 | Heaviness of head(头昏重) | Four-level scale |
| 4 | Blurred vision(眼花) | Four-level scale |
| 5 | Sleepiness(眼皮重) | Four-level scale |
| 6 | Asthenopia(视物疲劳) | Four-level scale |
| 7 | Red eye(目赤) | Four-level scale |
| 8 | Tinnitus(耳鸣) | Four-level scale |
| 9 | Epistaxis(鼻出血) | Four-level scale |
| 10 | Dryness of nasal cavity(鼻干) | Four-level scale |
| 11 | Dark purple lip and nail(唇甲紫暗) | Four-level scale |
| 12 | Bitter taste in mouth(口苦) | Four-level scale |
| 13 | Bland taste in mouth(口淡) | Four-level scale |
| 14 | Sticky and greasy sensation in mouth(口腻) | Four-level scale |
| 15 | Thirst(口干渴) | Four-level scale |
| 16 | Fetid mouth odor(口臭) | Four-level scale |
| 17 | Aphtha(口舌生疮) | Four-level scale |
| 18 | Gum bleeding(牙龈出血) | Four-level scale |
| 19 | Swelling in the gum(牙龈肿痛) | Four-level scale |
| 20 | Odontoseisis(牙齿松动) | Four-level scale |
| 21 | Dryness of the throat(咽喉干燥) | Four-level scale |
| 22 | Sore throat(咽喉肿痛) | Four-level scale |
| 23 | Sighing(太息) | Four-level scale |
| 24 | Yawn(呵欠) | Four-level scale |
| 25 | Belching(嗳气) | Four-level scale |
| 26 | Spontaneous sweating(自汗) | Four-level scale |
| 27 | Night sweating(盗汗) | Four-level scale |
| 28 | Palpitation(心悸) | Four-level scale |
| 29 | Stuffiness in chest(胸闷) | Four-level scale |
| 30 | Somnolence(嗜睡) | Four-level scale |
| 31 | Insomnia(失眠) | Four-level scale |
| 32 | Dreamfulness(多梦) | Four-level scale |
| 33 | Amnesia(健忘) | Four-level scale |
| 34 | Abdominal pain(腹痛) | Four-level scale |
| 35 | Abdominal distention(腹胀) | Four-level scale |
| 36 | Epigastric distending pain(胃脘胀痛) | Four-level scale |
| 37 | Epigastric dull pain(胃脘隐痛) | Four-level scale |
| 38 | Epigastric stabbing pain(胃脘刺痛) | Four-level scale |
| 39 | Acid swallow(吞酸) | Four-level scale |
| 40 | Gastric stuffiness(脘闷) | Four-level scale |
| 41 | Anorexia(纳差) | Four-level scale |
| 42 | Nausea and vomiting(恶心呕吐) | Four-level scale |
| 43 | Hiccup(呃逆) | Four-level scale |
| 44 | Lack of strength(倦怠乏力) | Four-level scale |
| 45 | Distending pain of hypochondrium(胁肋胀痛) | Four-level scale |
| 46 | Dull pain of hypochondrium(胁肋隐痛) | Four-level scale |
| 47 | Stabbing pain of hypochondrium(胁肋刺痛) | Four-level scale |
| 48 | Dysphoria and irritability(烦躁易怒) | Four-level scale |
| 49 | Lumbago(腰痛) | Four-level scale |
| 50 | Cold lumbus(腰冷) | Four-level scale |
| 51 | Soreness of waist(腰酸) | Four-level scale |
| 52 | Weakness of knees(膝软) | Four-level scale |
| 53 | Vexing heat in chest, palms and soles(五心烦热) | Four-level scale |
| 54 | Afternoon tidal fever(午后潮热) | Four-level scale |
| 55 | Borborygmus(肠鸣) | Four-level scale |
| 56 | Loose stool(便溏) | Four-level scale |
| 57 | Constipation(便秘) | Four-level scale |
| 58 | Fetid smelly stools(大便秽臭) | Four-level scale |
| 59 | Tenesmus(里急后重) | Four-level scale |
| 60 | More nocturia(夜尿清长) | Four-level scale |
| 61 | Dark urine(小便发黄) | Four-level scale |
| 62 | Yellow tint of sclera and skin(身目发黄) | Four-level scale |
| 63 | Sallow complexion(面色萎黄) | Four-level scale |
| 64 | Dimmish and darkish complexion(面色晦暗) | Four-level scale |
| 65 | Pale and lusterless complexion(面色白) | Four-level scale |
| 66 | Flushing of cheeks(面色红赤) | Four-level scale |
| 67 | Spider angioma(蜘蛛痣) | Four-level scale |
| 68 | Liver palms(肝掌) | Four-level scale |
| 69 | Pruritus(皮肤搔痒) | Four-level scale |
| 70 | Scaly skin(肌肤甲错) | Four-level scale |
| 71 | Purpuric skin(皮肤紫癜) | Four-level scale |
| 72 | Numbness of skins(肢体麻木) | Four-level scale |
| 73 | Heavy body(肢体困重) | Four-level scale |
| 74 | Arthralgia(关节疼痛) | Four-level scale |
| 75 | Seminal emission(男子遗精) | Four-level scale |
| 76 | hypermenorrhea(月经量多) | Four-level scale |
| 77 | hypomenorrhea(月经量少) | Four-level scale |
| 78 | hyperleukorrhea(带下量多) | Four-level scale |
| 79 | Foul leukorrhea(带下气臭) | Four-level scale |
| 80 | Tongue color(舌色) | 1(pale);  2(light red);  3(red);  4(crimson);  5(light purple);  6(dark purple) |
| 81 | Position of tongue color(舌色部位) | 1(whole);  2(partial) |
| 82 | Lustrous/withered tongue(荣枯) | -1(lustrous);  0(normal);  1(withered) |
| 83 | Tough/tender tongue(老嫩) | -1(tough);  0(normal);  1(tender) |
| 84 | Enlarged/thin tongue(胖瘦) | -1(enlarged);  0(normal);  1(thin) |
| 85 | Teeth-marked tongue(齿痕) | Two-level scale |
| 86 | Spotted tongue(点刺) | Two-level scale |
| 87 | Fissured tongue(裂纹) | Two-level scale |
| 88 | Tongue with ecchymosis(瘀斑) | Two-level scale |
| 89 | Thin/thick tongue fur(薄厚) | -1(thin);  0(normal);  1(thick) |
| 90 | Moist/less fluid/dry/rough tongue coating(润少津燥糙) | 1(moist);  2(less fluid);  3(dry);  4(rough) |
| 91 | Greasy/curdy tongue fur(腻腐) | -1(greasy);  0(normal);  1(curdy) |
| 92 | Less coating or uncoated tongue(少苔无苔) | -1(less coating);  0(normal);  1(uncoated tongue) |
| 93 | Peeling coating(花剥) | Two-level scale |
| 94 | Position of tongue fur(苔质部位) | 1(whole);  2(partial) |
| 95 | Tongue coating color(苔色) | 1(white);  2(yellowish);  3(yellow);  4(deep yellow);  5(grey);  6(dark) |
| 96 | Position of the color of tongue fur(苔色部位) | 1(whole);  2(partial) |
| 97 | Sublingual vein(舌下脉络) | Four-level scale |
| 98 | Floating/deep pulse(浮沉) | -1(floating);  0(normal);  1(deep) |
| 99 | Slow/rapid pulse(迟数) | -1(slow);  0(normal);  1(rapid) |
| 100 | Feeble/replete pulse(虚实) | -1(feeble);  0(normal);  1(replete) |
| 101 | Long/short pulse(长短) | -1(long);  0(normal);  1(short) |
| 102 | Wiry pulse(弦) | Two-level scale |
| 103 | Thready pulse(细) | Two-level scale |
| 104 | Slippery pulse(滑) | Two-level scale |
| 105 | Soggy pulse(濡) | Two-level scale |
| 106 | Moderate pulse(缓) | Two-level scale |
| 107 | Unsmooth pulse(涩) | Two-level scale |
| 108 | Weak pulse(弱) | Two-level scale |

Two-level scale: 0(negative); 1(positive)

Four-level scale: 1(normal); 2(slight); 3(medium); 4(serious)

**Table S1B. The definitions on tongue appearance indexes**

|  | **Feature** | | **Description** |
| --- | --- | --- | --- |
| tongue color (舌色) | pale tongue | 淡白舌 | a tongue less red than normal, indicating qi and blood deficiency or presence of deficiency-cold |
|  | pale red tongue | 淡红舌 | a tongue of normal color |
|  | red tongue | 红舌 | a tongue redder than normal, indicating presence of heat |
|  | crimson tongue | 红赤舌 | a tongue deep red in color, indicating intense heat |
|  | dark red tongue | 暗红舌 | a tongue darker and redder than crimson tongue, indicating extreme heat |
|  | bluish purple tongue | 淡紫舌 | a cyanotic tongue, indicating blood stasis or heat toxin in the nutrient-blood |
|  | purple tongue | 紫舌 | a tongue purple in color, indicating stagnant circulation of qi and blood |
| form of tongue (舌形) | flourishing tongue | 荣 | a luxuriant tongue is moistened and fresh red in color |
|  | withered tongue | 枯 | a withered tongue is dull, dark, dry and shriveled |
|  | tough tongue | 老 | a tough tongue is firm with rough texture, indicating excess syndromes |
|  | tender tongue | 嫩 | a tender tongue is delicate with fine texture, indicating deficiency syndromes |
|  | thin tongue | 瘦 | a tongue thinner than normal, indicating dual deficiency of qi and blood, or yin deficiency with effulgent fire |
|  | enlarged tongue | 胖 | a tongue that is larger than normal, indicating phlegm-fluid retention |
|  | teeth-marked tongue | 齿痕 | a tongue with dental indentations on its margin, indicating spleen deficiency or dampness encumbrance |
|  | spotted tongue | 点刺 | a tongue with red, white or black spots as well as thorn-like protrusions on its surface, indicating the heat toxin penetrating deeply in the blood aspect |
|  | fissured tongue | 裂纹 | a tongue with fissures on its surface, indicating yin-blood depletion |
|  | ecchymosis | 瘀斑 | a tongue with ecchymosis on its surface, indicating blood stasis |
| coating texture (苔质) | less fur | 少 | a tongue with less tongue coating |
|  | thin fur | 薄 | a tongue coating through which the underlying tongue surface is faintly visible, indicating the external contracted exterior syndrome or mild internal damage |
|  | thick fur | 厚 | a tongue coating through which the underlying tongue surface is not visible, indicating exterior pathogen entering the interior or phlegm-fluid retention and food accumulation |
|  | moist fur | 润 | a moderately moistened tongue coating, indicating the normal fluid |
|  | less fluid fur | 少津 | a less moistened tongue coating, indicating the less fluid damaging |
|  | dry fur | 燥 | a tongue coating that looks dry and feels dry to the touch, indicating the moderate fluid damaging |
|  | rough fur | 糙 | a tongue coating that looks like sand and feels rough sense, indicating severe exuberant heat damaging fluid |
|  | greasy fur | 腻 | a dense, turbid, slimy tongue coating, sticking on the tongue, hard to wipe off, indicating dampness turbidity, phlegm-fluid retention, or food accumulation |
|  | curdy fur | 腐 | a tongue coating consisting of coarse granules like bean dregs, easy to wipe off, indicating food accumulation or phlegm turbidity |
|  | peeling fur | 花剥 | complete or partial peeling of the tongue coating, indicating stomach qi and yin deficiency |
|  | without fur | 无苔 | a completely smooth tongue free of coating, indicating severe stomach yin deficiency |
| tongue coating color (苔色) | white fur | 白 | tongue coating white in color, indicating exterior syndrome or cold syndrome |
|  | yellowish fur | 微黄 | tongue coating light yellow in color, indicating mild interior syndrome or heat syndrome |
|  | yellow fur | 黄 | tongue coating yellow in color, indicating moderate interior syndrome or heat syndrome |
|  | dark yellow fur | 深黄 | tongue coating dark yellow in color, indicating severe interior syndrome or heat syndrome |
|  | gray fur | 灰 | tongue coating gray in color with similar clinical significance as black fur |
|  | black fur | 黑 | tongue coating black in color, indicating either excessive cold or extreme heat in the interior |
|  | sublingual vein | 舌下脉络 | the vein under the tongue on either side of the frenulum |

**Table S1C. The definitions on pulse indexes**

| **Feature** | | **Description** |
| --- | --- | --- |
| floating pulse | 浮 | a superficially located pulse which can be felt by light touch and grows faint on hard pressure |
| deep pulse | 沉 | a deeply located pulse which can only be felt when pressing hard |
| slow pulse | 迟 | a pulse with less than four beats to one cycle of the physician’s respiration, the same as bradycardia |
| rapid pulse | 数 | a pulse with more than five or six beats to one cycle of the physician’s respiration, the same as tachycardia |
| feeble pulse | 虚 | a feeble and void pulse |
| replete pulse | 实 | a pulse felt forceful at all the three sections, cun/inch, guan/bar and chi/cubit, also called forceful pulse |
| long pulse | 长 | a pulse with beats of long duration, exceeding cun/ inch, guan/bar and chi/cubit sections |
| short pulse | 短 | a pulse with beats of short duration, only felt at guan/ bar section |
| wiry pulse | 弦 | a straight, long and taut pulse, like a musical string to the touch |
| thready pulse | 细 | a pulse as thin as a silk thread, straight and soft, feeble yet always perceptible upon hard pressure, also called thin/thready pulse |
| slippery pulse | 滑 | a pulse coming and going smoothly like beads rolling on a plate |
| soggy pulse | 濡 | a thin and floating pulse which can be felt on light pressure, but growing faint upon hard pressure |
| moderate pulse | 缓 | a pulse with four beats to one cycle of the physician’s respiration, even and harmonious in its form |
| unsmooth pulse | 涩 | a pulse coming and going unsmoothly with small, fine, slow joggling tempo like scraping bamboo with a knife |
| weak pulse | 弱 | a pulse that is deep, soft, thin and forceless |

Table S2. Modern medicine features.

| **Index** | **Feature** | **Data range** |
| --- | --- | --- |
| 1 | TBIL | 3.9~267.3 umol/L |
| 2 | DBIL | 1.0~220.9 umol/L |
| 3 | IDBIL | 0.0~64.8 umol/L |
| 4 | ALT | 10~804 IU/L |
| 5 | AST | 14~336 IU/L |
| 6 | GGT | 1~634 IU/L |
| 7 | ALP | 13~290 IU/L |
| 8 | TP | 47~86 g/L |
| 9 | ALB | 9~65 g/L |
| 10 | PreAlb | 57~1384 mg/L |
| 11 | bile acid | 2~390 umol/L |
| 12 | PT | 8.6~25.6 s |
| 13 | TT | 10.7~28.0 s |
| 14 | APTT | 14.9~62.3 s |
| 15 | HBsAg | 0~250 IU/ml |
| 16 | HBsAb | 0~450.99 mIU/ml |
| 17 | HBeAg | 0.03~1592.82 S/CO |
| 18 | HBeAb | 0~65.19 S/CO |
| 19 | HBcAb | 0.05~21.47 S/CO |
| 20 | HbcAbIgM | 0~36 S/CO |
| 21 | Pre-S1Ag | Two-level scale A |
| 22 | Pre-S1Ab | Two-level scale A |
| 23 | T3 | 0.3~1350 nmol/L |
| 24 | RT3 | 0.44~3.95 nmol/L |
| 25 | T4 | 5.4~320 nmol/L |
| 26 | T | 0.09~52.05 nmol/L |
| 27 | E2 | 1.79~2402 pmol/L |
| 28 | bilirubin | Four-level scale A |
| 29 | bilirubin 2nd | Two-level scale A |
| 30 | occult blood | Four-level scale A |
| 31 | red corpuscle(microscope) | Four-level scale B |
| 32 | glucose(urine) | Two-level scale A |
| 33 | glucose(urine) 2nd | Four-level scale A |
| 34 | acetone body | Four-level scale A |
| 35 | acetone body 2nd | Two-level scale A |
| 36 | granular cast | Two-level scale A |
| 37 | leucocyte | Four-level scale B |
| 38 | leucocyte(microscope) | Four-level scale B |
| 39 | nitrite | Four-level scale A |
| 40 | PH value | 0~9 |
| 41 | protein | Four-level scale A |
| 42 | protein 2nd | Two-level scale A |
| 43 | specific gravity | 0~7 |
| 44 | epithelial cell | Four-level scale B |
| 45 | hyaline cylinder | Two-level scale A |
| 46 | urobilinogen | Four-level scale A |
| 47 | urobilinogen 2nd | Two-level scale A |
| 48 | CD3 value | 0~2.93 % |
| 49 | CD4 value | 0~1.66 % |
| 50 | CD8 value | 0.03~1.38 % |
| 51 | IgA | 0.69~18.4 g/L |
| 52 | IgM | 0.32~20.6 g/L |
| 53 | IgG | 0.56~42.5 g/L |
| 54 | BUN | 2.1~11.3 mmol/L |
| 55 | Cr | 25~180 umol/L |
| 56 | uric acid | 102~649 umol/L |
| 57 | glucose | 2.38~12.05 mmol/L |
| 58 | TC | 1.93~11.24 mmol/L |
| 59 | TG | 0~6.03 mmol/L |
| 60 | HDLC | 0.21~3.65 mmol/L |
| 61 | LDLC | 0.43~7.58 mmol/L |
| 62 | APOA1 | 0~3.17 g/L |
| 63 | albumin | 23.8~53.79 % |
| 64 | α1-MG | 0.36~4.40 % |
| 65 | α2-MG | 2.5~10.03 % |
| 66 | β-MG | 0.90~12.46 % |
| 67 | γ-MG | 1.64~27.78 % |
| 68 | AFP | 0.62~9687 ng/ml |
| 69 | HA | 1.3~800 ug/L |
| 70 | LN | 9.9~283.6 ug/L |
| 71 | Ⅳ－C | 3.3~147.4 ug/L |
| 72 | PⅢP | 1.11~6.4 nmol/L |
| 73 | basophilic granulocyte | 0~0.55 10^9^/L |
| 74 | basophilic granulocyte_A | 0~2.77 % |
| 75 | eosinophilic granulocyte | 0~1.4 10^9^/L |
| 76 | eosinophilic granulocyte_A | 0~20.63 % |
| 77 | hematocrit | 0.16~50.8 |
| 78 | hemoglobin | 89~192 g/L |
| 79 | lymphocyte | 0.62~4.13 10^9^/L |
| 80 | lymphocyte_A | 1.57~67.24 % |
| 81 | mean corpuscular hemoglobin | 19~39.4 pg |
| 82 | mean corpuscular hemoglobin concentration | 108~407 g/L |
| 83 | mean corpuscular volume | 32~103.1 fL |
| 84 | monocyte | 0~1.86 10^9^/L |
| 85 | monocyte_A | 0~44 % |
| 86 | mean platelet volume | 0~25.6 fL |
| 87 | neutrophilic granulocyte | 0.02~14 10^9^/L |
| 88 | neutrophilic granulocyte_A | 0.18~85.3 % |
| 89 | thrombocytocrit | 0~32.7 % |
| 90 | blood platelet dispersion of distribution | 0~23.9 fL |
| 91 | blood platelet | 38~486 10^9^/L |
| 92 | red corpuscle | 3.19~7.07 10^12^/L |
| 93 | red corpuscle dispersion of distribution | 1.5~42.2 fL |
| 94 | leucocyte_A | 1.54~15.3 % |
| 95 | red corpuscle_A | Two-level scale A |
| 96 | leucocyte_B | Two-level scale A |
| 97 | consistency of stools | 1(watery);  2(soft);  3(hard) |
| 98 | color of stools | 1(yellow);  2(clay colored);  3(black);  4(brown);  5(tan);  6(green-yellow) |
| 99 | liver surface | 1(smooth);  2(rough);  3(very rough);  4(hackly) |
| 100 | Liver edge | 1(sharp);  2(blunt) |
| 101 | density of the echo | 1(normal);  2(slightly enhanced);  3(significantly enhanced) |
| 102 | intensity distribution | 1(even);  2(uneven);  3(tuberous) |
| 103 | vascular texture | 1(clear);  2(vague);  3(very vague);  4(narrow);  5(uneven thickness) |
| 104 | length of the left hepatic lobe | 18~119 mm |
| 105 | thickness of the left hepatic lobe | 8~108 mm |
| 106 | the largest oblique diameter of the left hepatic lobe | 56~182 mm |
| 107 | inner diameter of portal | 8~14 mm |
| 108 | Length | 7.2~182 mm |
| 109 | Thickness | 2.7~95 mm |
| 110 | subcostal length | 0~138 mm |
| 111 | inner diameter of vena lienalis | 0.6~17 mm |
| 112 | gall bladder condition | Two-level scale B |
| 113 | Kidney condition | Two-level scale B |
| 114 | Classification of liver condition by integral evaluation | 1(smooth surface, sharp edge, clear vessel);  2(smooth surface, enhanced echo, clear vessel)  3(rough surface, blunt edge, enhanced and uneven echo, vague vessel)  4(very rough surface, significantly enhanced and uneven echo, very vague vessel)  5(hackly surface, significantly enhanced, uneven and nodular echo, narrow or uneven thickness vessel) |

Two-level scale A: 1(negative); 2(positive)

Two-level scale B: 1(normal); 2(abnormal)

Four-level scale A: 1(negative,+-); 2(+); 3(++); 4(+++)

Four-level scale B: 1(0-5); 2(5-9); 3(10-19); 4(15-20); 5(>20) Cells/HP

**Text S1. A random forest based co-training algorithm for TCM syndrome prediction**

Given a set *L* of labeled examples and a set *U* of unlabeled examples, the examples are represented with two views of features as *X* and *Z*, the basic co-training algorithm first creates a smaller pool *U’* containing *u* unlabeled examples. It then iterates the following procedure. (1) Use *L* to train two distinct classifiers *h_1_* and *h_2_* which are classifiers based on the *X* and *Z* respectively. (2) Allow each of these two classifiers to examine the unlabeled set *U’* and select the *p* examples it most confidently labels as positive, and the *n* examples it most confidently labels negative. Each example selected in this way is added to *L*, along with the label assigned by the classifier that selected it. (3) The pool *U’* is replenished by drawing 2*p*+2*n* examples from *U* at random.

In this study, the random forests (RF) procedure was adopted as the base classifier in the co-training schema for following reasons. (1) RF avoid to perform feature selection which commonly induce more computational complexity, since at each step of RF tree construction only a small randomly selected subset of the variables is used to construct the partition. (2) RF overcomes over-fitting by taking the Bagging technique . Each observation is predicted by each tree in the forest and its final predicted label corresponds to the majority-voted class, provides robustness and improved accuracy . (3) RF provides the efficient assessment of variable importance contributed to different views (TCM view and modern medicine view in our study).


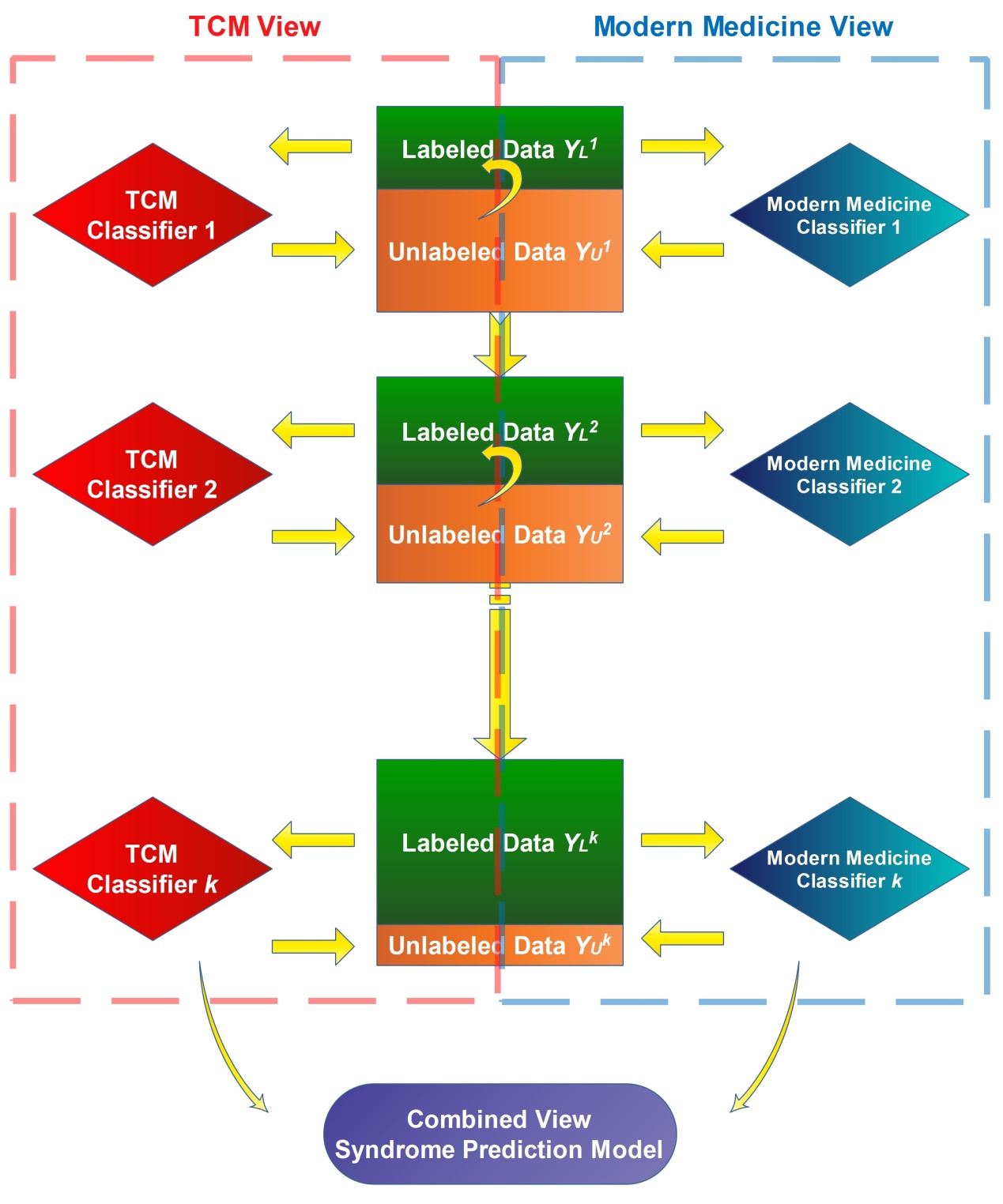


**Figure S1.** The procedure of the co-training algorithm used in this study. Several parameters should be set before training, *i.e.*, the size of labeled data, the preference parameter *c* and the iteration parameter.

It should be noted that we also improved the traditional version of co-training schema to solve our multiple class and data unbalanced issues. A general pipeline of our co-training algorithm is described in Figure S1, in which TCM view and modern medicine view are considered as *X* and *Z* measurements respectively. Different from the traditional version of co-training which assumes the labeled data is much smaller than the unlabeled data, the subset *U’* is no longer used for the initialization step in this work. Thus, the first step is training the classifier for both the *X* measurements and *Z* measurements (*X* and *Z* are the two views) with labeled data. We set *Y_L_* the class variable of matrices *X_L_* and *Z_L_* (*L* stands for the labeled data, while *U* for unlabeled data). The classifiers used to predict either an observation *x* in *X* or in *Z* are denote by:

$\left\{\phi\left(X_{L}\left(x_{i}\right)\right),\phi\left(Z_{L}\left(z_{i}\right)\right)\right\}$ (1)

It should be noted that *L* in the above notation indicates that the classifier was trained using *X_L_* data with response *Y_L_* or *Z_L_* data with response *Y_L_*. The second step is giving the most confident classification for each observation *i* in *L*∪*U*. Subsequently, *φ*(*X*(*x_i_*),*Z*(*z_i_*)) is defined as a new classifier that classifies each observation in accordance with the most confident classification:

$\phi\left(X\left(x_{i}\right),Z(z_{i})\right)=\left\{\begin{matrix} \phi\left(X_{L}\left(x_{i}\right)\right),P\left(X\right)\rangle P\left(Z\right) \\ \phi\left(Z_{L}\left(z_{i}\right)\right),P\left(X\right)\langle P\left(Z\right) \end{matrix}\right.$ (2)

where

$P\left(X\right)=\max\left\{P\left(Y_{i}=1|X\right),P\left(Y_{i}=0|X\right)\right\}$ (3)

$P\left(Z\right)=\max\left\{P\left(Y_{i}=1|Z\right),P\left(Y_{i}=0|Z\right)\right\}$ (4)

In the third step, several specific unlabeled samples are chosen to add to the labeled group in the next iteration. In order to handle the situation that the classes are heavily unbalanced, the selecting rules are designed as follows: a parameter *c* which reflects the selection preference of the class is involved for users to set. If *c* is set to 1, only the observers in unlabeled set which are classified as 1 by both the classifiers (*Y*=1 | *X*, *Z*) are added to labeled set for the next iteration, while the ones classified as 0 (*Y*=0 | *X*, *Z*) are added to labeled set when *c*=0. In addition, *c* is allowed to be set to NA, in which case all the observers with the same prediction result by the two classifiers can be added to the labeled group.

Therefore, in iterative phase, the training set form in the *i*th iteration when *c* is set to 0 for instance is defined as

$Y^{i}=\left(\begin{matrix} Y_{L}^{i-1} \\ P^{i-1}\left(Y_{U}^{i-1}=0|X,Z\right) \end{matrix}\right)$ (5)

*L* and *U* mean labeled and unlabeled dataset respectively in formula (5). The procedure then performs the first step again to generate {*φ*(*X*(*x_i_*)),*φ*(*Z*(*z_i_*))} and this time the classifiers were generated with class variable *Y_i_* and the full data matrices *X*, *Z*. This phase is repeated for *k*=2, … , *k* times using training class variables *Y_2_*, … , *Y_k_*. Upon exiting the algorithm after *k* iterations, the model provides an estimated class for each observation in the data. In addition, the final classifier can be used to directly predict new observations unavailable at the time of training.

This proposed co-training algorithm can also produce variable importance whenever RF is used in the classifiers. To incorporate this variable importance, each observation in *L*∪*U* is taken into consideration. Let *i_k_* be the view indicator such that *i_k_*=1 if the observation *k* is from view one, then the variable importance for variable *j* in view *v* is defined as:

$VS_{j}=\sum_{k=1}^{L\cup U} S_{kj}\left(X_{v}\right)1_{\left\{i_{k}=v\right\}}$ (6)

where *VS* stands for Variable Score, and *S_kj_*(*X_v_*) is the variable score for observation *k*. To obtain a variable ranking from the co-training algorithm, the variable scores are concatenated and sorted with high positive values being most important.

1. S. Caetano, B. Ustun, S. Hennessy, J. Smeyers-Verbeke, W. Melssen, G. Downey, L. Buydens, Y. Heyden, Geographical classification of olive oils by the application of CART and SVM to their FT-IR. *J. Chemometrics.* **21**, 324 (2007).

2. L. Breiman, Random forests. *Machine Learning.* **45**, 5 (2001).
